# Ultralow-Loading Anthrone Molecular Semiconductor for Enhancing the Insulation Reliability of Silicone Gel Dielectrics

**DOI:** 10.3390/gels12080668

**Published:** 2026-07-25

**Authors:** Mengjia Feng, Chaoyue Zhao, Wenbo Li, Zichen Cui, Jianzeng Guo

**Affiliations:** 1 State Key Laboratory of Smart Power Distribution Equipment and System, Hebei University of Technology, Tianjin 300123, China; 202431402163@stu.hebut.edu.cn (C.Z.); 202531402049@stu.hebut.edu.cn (W.L.); 202531402010@stu.hebut.edu.cn (Z.C.); 202511401012@stu.hebut.edu.cn (J.G.); 2 School of Electrical Engineering, Hebei University of Technology, Tianjin 300123, China

**Keywords:** silicone gel, anthrone, deep traps, insulation strength

## Abstract

Silicone gel (SG) is an important soft encapsulation dielectric for high-voltage power modules, yet its limited insulation performance under high electric fields and elevated temperatures restricts its practical application. Herein, an ultralow loading of the organic molecular semiconductor anthrone (ET) was introduced into silicone gel to simultaneously improve dielectric properties and thermal stability. SG-ET_0.5_ exhibited the best overall performance, with a breakdown strength of 29.14 kV/mm at 25 °C, 19.57% higher than that of pristine SG, and retained 22.86 kV/mm at 150 °C with only a 21.56% reduction. The relative permittivity increased to 3.16 and 2.85 at 25 °C and 200 °C, respectively. The partial discharge inception voltage increased from 3.1 to 4.8 kV, while both discharge frequency and amplitude were markedly reduced. Moreover, SG-ET_0.5_ showed an increased 5% weight-loss temperature of 370 °C, together with slightly increased thermal conductivity and a reduced coefficient of thermal expansion. Mechanistic analysis suggests that the low-lying LUMO level and molecular characteristics of ET may contribute to the increased deep-trap density and enhanced electron-capturing tendency of the composites, thereby helping to suppress electron avalanche development and partial discharge. This work offers a molecular-level strategy for improving the electrical insulation performance of silicone gel dielectrics for high-voltage power modules.

## 1. Introduction

High-voltage power electronic devices have been widely used in extra-high-voltage flexible DC transmission, new energy vehicles, aerospace systems, and other advanced engineering fields. Currently, wide-bandgap (WBG) semiconductor materials, such as SiC and GaN, are driving power modules toward higher operating voltages, higher power densities, and harsher thermal environments owing to their high breakdown strength, high thermal conductivity, and high operating-temperature capability [1,2]. These developments impose unprecedented requirements on the electrical and thermal performance and stability of encapsulation insulating materials [3,4,5,6,7]. Silicone gel (SG), which combines excellent heat resistance, electrical insulation, and mechanical compliance, has become one of the most promising soft encapsulation dielectrics for high-voltage power modules. Its unique flexibility enables effective mitigation of thermomechanical mismatch stress in rigid packaging systems [8,9,10,11]. Nevertheless, silicone gel remains susceptible to electrical deterioration under high electric fields. In particular, localized electric-field distortion at the triple-junction region composed of the ceramic substrate, metal conductor, and gel can promote charge injection and partial-discharge activity, thereby increasing the risk of local insulation failure [12,13,14,15,16].

For soft encapsulation dielectrics such as silicone gel, enhancing dielectric strength while maintaining fluidity and processability remains a typical performance trade-off. Conventional strengthening strategies usually rely on suppressing carrier injection, migration, and accumulation by increasing crosslinking density, introducing inorganic fillers, or constructing denser molecular networks. However, these approaches often increase viscosity, reduce flowability, and deteriorate interfacial adaptability, which may compromise the key advantages of silicone gel in mitigating thermomechanical mismatch stress and accommodating complex packaging architectures. Therefore, regulating charge transport and improving the intrinsic insulation strength of silicone gel without sacrificing potting processability remain critical challenges for developing highly reliable insulating materials for power packaging [17]. From a material-selection perspective, identifying an optimal silicone-gel formulation is inherently a multi-criteria engineering problem because electrical insulation, thermal stability, mechanical compliance, flowability, and processing adaptability may represent competing objectives. Multi-criteria ranking and rank-aggregation methods can provide systematic frameworks for integrating these competing performance criteria and identifying formulations with balanced overall properties [18,19].

Existing strategies for enhancing the performance of silicone gel mainly fall into two categories: inorganic filler incorporation and molecular structure modification [20]. Inorganic filler incorporation improves thermal and electrical properties by constructing thermally conductive pathways, enhancing nonlinear conductivity, or introducing deep traps [21,22,23,24,25,26,27]. For instance, Wang et al. introduced deep traps into polymers by incorporating POSS fillers, thereby suppressing charge transport and electrical tree growth [25]. Luo et al. enhanced thermal performance without sacrificing electrical properties by introducing SiO_2_/nanodiamond hybrids [26]. Sun et al. improved thermal management performance by incorporating graphene derivatives, although this was accompanied by a decrease in insulation performance [27]. However, inorganic filler incorporation usually requires relatively high filler loadings, which can increase system viscosity and reduce flowability, thereby seriously affecting the practical encapsulation process. In addition, filler agglomeration and interfacial defects may induce local electric-field concentration, further weakening insulation reliability [28,29]. Molecular structure modification can regulate dielectric response and breakdown behavior at the molecular scale by introducing functional groups such as phenyl or trifluoropropyl groups [30,31]. Nevertheless, this approach often suffers from complex synthetic routes, side reactions, and limited process controllability. Overall, existing methods still struggle to simultaneously achieve low additive loading, effective insulation enhancement, and good encapsulation processability. Therefore, an efficient and facile molecular design strategy for enhancing the insulation performance of SG remains highly desirable.

In recent years, studies on polymer dielectrics have shown that organic molecular semiconductors with high electron affinity can be introduced into polymer matrices such as PEI. These molecules usually possess conjugated structures and strong electron-trapping capability, enabling the formation of deep trap levels in the matrix. As a result, injected carriers can be effectively captured, carrier mobility can be reduced, and leakage current increase and charge accumulation under high electric fields or elevated temperatures can be suppressed [32,33,34]. However, most existing studies have focused on rigid or film-type polymer dielectrics, and their applicability to silicone gel remains unclear. For silicone gel with low crosslinking density, high chain-segment mobility, and complex service interfaces, it is still unknown whether organic molecular semiconductors can effectively improve intrinsic insulation properties without significantly altering gel flowability and processing adaptability. Moreover, their effects on high-temperature breakdown strength, partial-discharge tolerance, and thermal stability remain insufficiently understood.

Inspired by this, an ultralow-loading organic molecular semiconductor modulation strategy was proposed in this work. Anthrone (ET), as an organic molecular semiconductor, was introduced into silicone gel to enhance insulation performance and thermal stability through deep-trap regulation. The microstructural evolution, thermal behavior, and electrical insulation properties of silicone gel composites with different anthrone contents were systematically investigated, and the mechanism underlying the organic molecular semiconductor-enabled insulation enhancement of silicone gel was revealed. This work opens a new route for designing advanced encapsulation dielectrics that integrate mechanical compliance, thermal stability, and high dielectric strength.

## 2. Results and Discussion

### 2.1. Microstructural Morphology of SG-ET Composites

**Figure 1:**
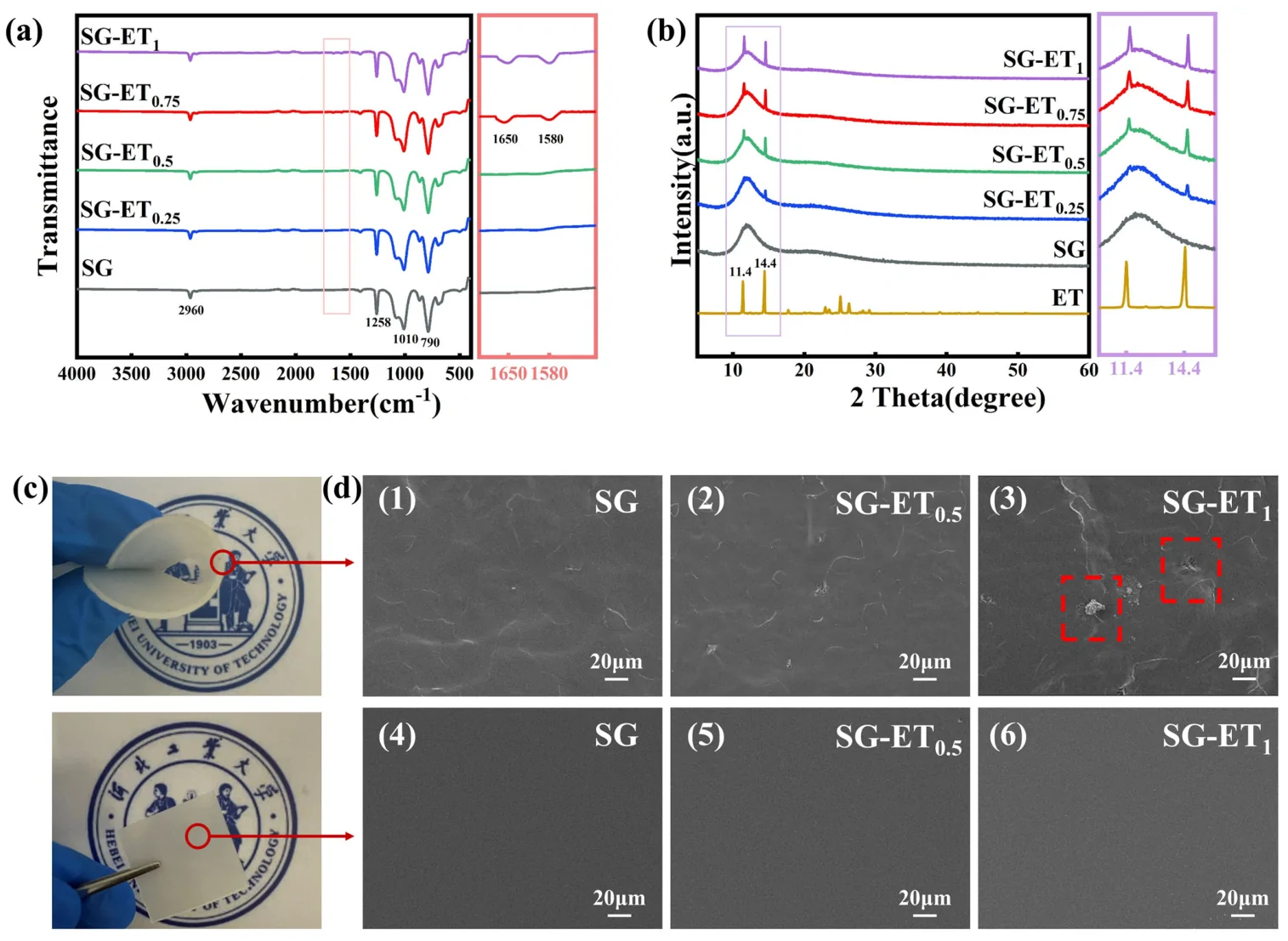
Structural and morphological characterization of SG-ET composites. (**a**) FTIR spectra of samples with different anthrone contents. (**b**) XRD patterns of samples with different anthrone contents and anthrone. (**c**) Schematic illustration of silicone gel composite samples. (**d**) SEM images of samples with different anthrone contents at a 20 μm scale bar; images (**d1**–**d3**) show cross-sectional morphologies, and images (**d4**–**d6**) show surface morphologies.

To characterize the molecular structure of silicone gel and evaluate the influence of ET incorporation on the polymer network, FTIR spectra of SG and SG-ET composites were collected, as shown in Figure 1a. For pristine SG, the characteristic absorption peaks located at 2960, 1258, 1010, and 790 cm^−1^ were assigned to the C-H stretching vibration of Si-CH_3_, the in-plane C-H bending vibration, the Si-O-Si stretching vibration, and the Si-C stretching vibration, respectively, indicating the formation of a typical silicone gel network structure [35]. Owing to the ultralow ET loading, the spectra of the composites remained dominated by the characteristic absorption bands of the silicone gel matrix, and no obvious changes in the positions or intensities of these bands were observed. When the ET content reached 0.75 wt% and above, weak additional absorption bands became discernible near 1650 and 1580 cm^−1^, which are consistent with the C=O stretching vibration and aromatic C=C skeletal vibration of ET, respectively. At lower ET contents, including SG-ET_0.5_, these weak ET-related bands cannot be clearly resolved because of the ultralow ET concentration and the dominant absorption of the silicone gel matrix. This limited spectral resolution does not indicate the absence of ET-related carbonyl groups. The absence of new prominent absorption bands or systematic shifts in the silicone-gel-related peaks further suggests that ET incorporation did not substantially alter the primary crosslinked network of silicone gel.

To further analyze the effect of ET on the microstructure of silicone gel, XRD patterns of pristine ET and SG-ET composites with different ET contents were collected, as shown in Figure 1b. Pristine ET exhibited distinct crystalline characteristics, with two main diffraction peaks located at 11.42° and 14.44°. In contrast, pristine SG showed only a broad diffuse diffraction halo centered at approximately 12°, reflecting its typical amorphous siloxane network. With increasing ET content, the ET-related reflections became gradually more discernible in the SG-ET composites and exhibited slight shifts toward higher 2θ values. For SG-ET_1_, the two peaks were located at 11.58° and 14.58°, corresponding to right shifts of 0.16° and 0.14°, respectively, relative to pristine ET. These shifts toward higher diffraction angles suggest a slight decrease in the corresponding intermolecular spacing, which may be associated with a minor adjustment of the local packing environment of ET after incorporation into the silicone gel matrix. Meanwhile, the broad diffuse profile of SG remained essentially unchanged. These results suggest that ET may retain or develop a certain degree of localized crystalline ordering after incorporation into the silicone gel matrix, particularly at higher ET loadings, while the amorphous network structure of silicone gel was largely preserved. Together with the weak ET-related FTIR bands, the XRD results are consistent with the possible presence of localized ET-rich regions or microcrystalline ordering in the silicone gel matrix. The absence of substantial changes in the characteristic FTIR bands and amorphous XRD profile of silicone gel suggests that ET was predominantly physically incorporated without obvious reconstruction of the silicone gel network.

SEM was further employed to examine both the cross-sectional and surface microstructures of the samples, as presented in Figure 1d. All samples exhibited relatively smooth surfaces, suggesting good macroscopic homogeneity after curing. The cross sections of pristine silicone gel and low-ET-content composites showed relatively uniform morphologies, with only a few shallow cracks and isolated discontinuities, indicating that limited anthrone incorporation did not significantly disturb the continuity of the silicone gel network. However, as the anthrone content increased, the fracture surfaces became rougher and more heterogeneous. Notably, SG-ET_1_ exhibited more evident granular protrusions, interfacial discontinuities, and microporous regions. These morphological features may be associated with localized anthrone enrichment or aggregation, which could reduce interfacial continuity and introduce additional structural defects. Such structural inhomogeneity may adversely affect the dielectric performance of the composites by introducing local defects and electric-field inhomogeneity.

### 2.2. Physical Characterization of SG-ET Composites

**Figure 2:**
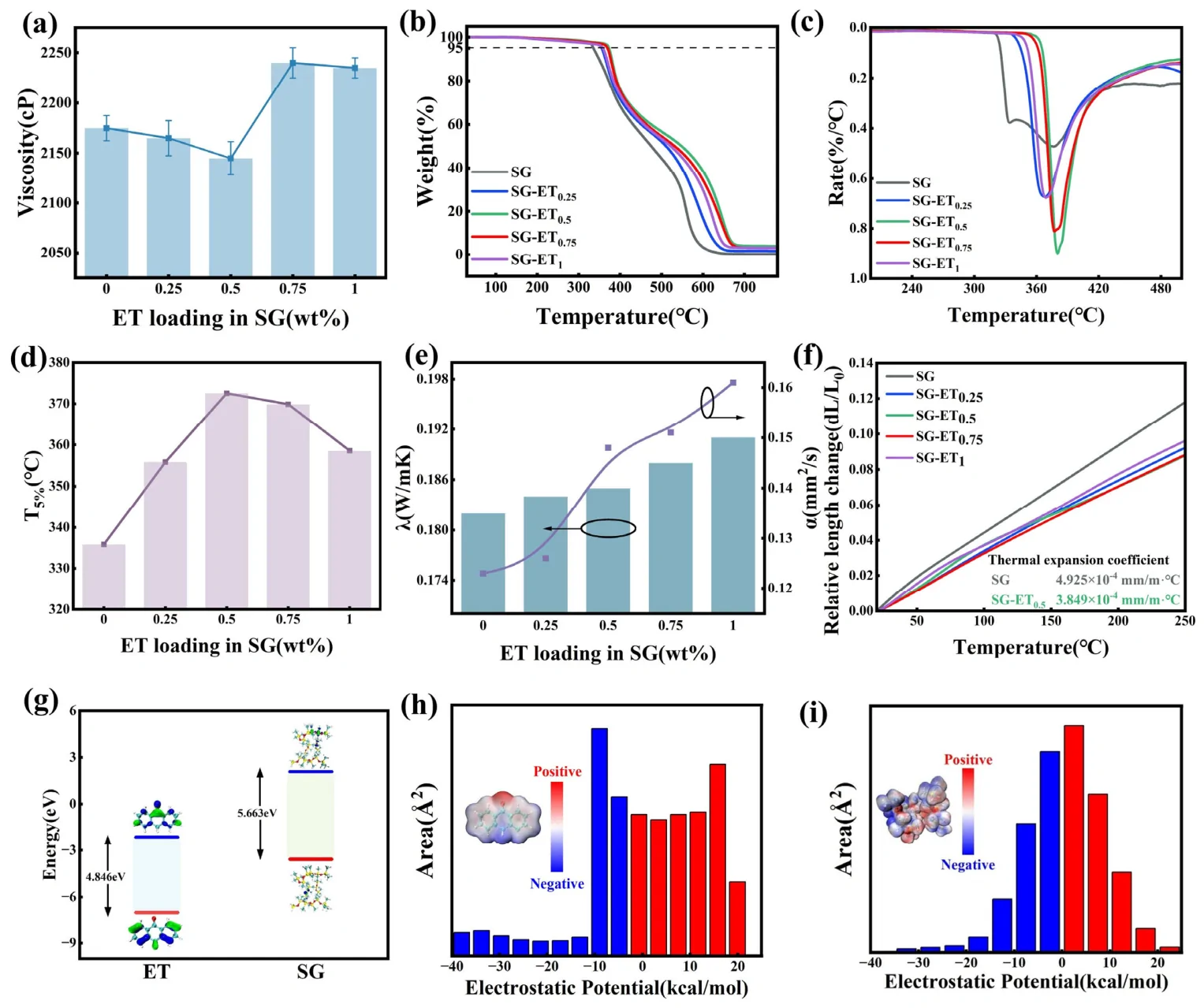
(**a**) Viscosity of the composite solutions before curing. (**b**) TGA curves of samples with different ET contents. (**c**) DTG curves of samples with different ET contents. (**d**) Temperature at 5% weight loss of samples with different ET contents. (**e**) Thermal conductivity of samples with different ET contents. (**f**) Thermal diffusivity of samples with different ET contents. (**g**) Energy band diagram of the SG and ET molecular models. (**h**) Electrostatic potential distribution and normalized surface area of ET. (**i**) Electrostatic potential distribution and normalized surface area of SG.

For potting insulating materials, good flowability is a prerequisite for defect-free encapsulation. To evaluate the effect of ET incorporation on the processability of silicone gel, the viscosity of uncured SG-ET composite mixtures was measured at 25 °C, as shown in Figure 2a. The initial viscosity of pristine SG was approximately 2175 cP, and only slight fluctuations were observed with increasing ET content, indicating that the introduction of an ultralow loading of ET did not significantly affect the flowability of silicone gel. SG-ET_0.5_, which exhibited the best insulation performance, showed a viscosity of approximately 2145 cP, almost identical to that of pristine SG, suggesting its good compatibility with the potting encapsulation process. When the ET content increased to 0.75 wt%, the viscosity slightly increased, which may be associated with possible localized ET enrichment or microcrystalline ordering at higher concentrations and the resulting perturbation of the rheological behavior of the system.

From the TGA curves in Figure 2b and the DTG curves in Figure 2c, no obvious mass loss was detected for any sample below 300 °C, suggesting that all silicone gel-based systems possessed good resistance to initial thermal degradation. After ET incorporation, the thermal decomposition process of the composites shifted toward higher temperatures. Among all samples, the thermal degradation of SG-ET_0.5_ was delayed most effectively, which was confirmed by the higher onset mass-loss temperature and DTG peak temperature. As shown in Figure 2d, SG-ET_0.5_ showed the highest 5% weight-loss temperature (T_5%_) of 370 °C, suggesting that an appropriate amount of ET effectively delayed the initial thermal degradation of silicone gel. With further increasing temperature, pristine SG showed a significantly faster weight-loss rate than the composites, whereas all SG-ET composites maintained better high-temperature stability. These results indicate that ET incorporation enhanced the thermal resistance of SG to some extent. This improvement may be attributed to the rigid aromatic conjugated structure of ET molecules [36], which can restrict the thermal motion of polymer chain segments and delay the early decomposition of the matrix during heating.

To assess the influence of ET incorporation on heat transport, the thermal conductivity and thermal diffusivity of SG-ET composites were measured, as shown in Figure 2e. The thermal conductivity of pristine SG was approximately 0.182 W/m·K and increased slightly to 0.191 W/m·K with increasing ET content. Meanwhile, the thermal diffusivity showed a similar upward trend, increasing from 0.123 mm^2^/s for pristine SG to 0.161 mm^2^/s for SG-ET_1_. Overall, ET incorporation improved the heat transfer capability of the silicone gel system to some extent, although the enhancement was relatively limited. This moderate improvement may be attributed to two factors. On the one hand, the rigid aromatic conjugated structure of ET molecules is favorable for localized phonon transport. On the other hand, owing to the ultralow ET loading, with a maximum content of only 1 wt%, continuous heat-conduction pathways could hardly be formed within the silicone matrix, resulting in only a marginal increase in thermal conductivity. Notably, thermal diffusivity exhibited a more pronounced increase than thermal conductivity, suggesting that ET incorporation may preferentially improve the localized thermal response and heat diffusion rate. Therefore, ET had a positive but moderate effect on the thermal transport performance of silicone gel, while its more important role may lie in regulating the electrical insulation performance.

The coefficient of thermal expansion (CTE) is particularly important for encapsulation insulating materials used in power modules. A lower CTE helps reduce thermal expansion mismatch between silicone gel and other module components, such as chips, ceramic substrates, and metal electrodes, thereby alleviating interfacial thermomechanical stress concentration during thermal cycling and reducing the risks of encapsulation cracking, interfacial debonding, and local electric-field distortion [37]. Figure 2f shows the thermal expansion behavior of SG composites with different ET contents, and the CTE values were obtained by linear fitting. The CTE of pristine SG was 4.925 × 10^−4^ mm/m·°C, whereas that of SG-ET_0.5_ decreased to 3.849 × 10^−4^ mm/m·°C. With further increasing ET content, the CTE slightly increased again. The decrease in CTE suggests that moderate ET incorporation improved the ability of silicone gel to resist thermal expansion under thermal-field conditions. This improvement may be attributed to the intermolecular interactions formed between ET molecules and silicone gel molecular chains through the rigid aromatic structure and polar carbonyl groups of ET. These interactions can restrict the thermal motion of polysiloxane chain segments during heating, thereby reducing the macroscopic thermal expansion of the material. At higher ET contents, possible localized ET enrichment or nonuniform intermolecular interactions may weaken the restriction of silicone chain-segment motion, which could account for the less pronounced reduction in CTE. The lowest CTE of SG-ET_0.5_ indicates its superior thermal matching capability, which may help minimize thermomechanical stress in power modules.

### 2.3. Molecular Simulation Analysis

To compare the intrinsic electronic characteristics of ET and SG, DFT calculations were performed using Gaussian 16W A.03. An isolated ET molecule and a representative silicone gel chain segment were optimized separately using the B3LYP functional. The frontier molecular orbital energy-level distributions of ET and SG are shown in Figure 2g. The HOMO–LUMO energy gaps of the ET and SG molecular models were calculated to be 4.846 and 5.663 eV, respectively. The LUMO energy level of ET was substantially lower than that of SG, indicating its stronger intrinsic electron-accepting tendency. According to the molecular electron-trap model reported in the literature [38], the electron-trap level introduced by ET was estimated from the LUMO-level difference between the separately calculated SG and ET molecular models, *E_LUMO,SG_*—*E_LUMO,ET_*, yielding a value of 5.6604 eV. This energy-level relationship suggests that ET ay act as an electron-accepting component when dispersed in the silicone gel matrix. Owing to its low LUMO level, ET may provide energetically favorable states for electron capture and thereby contribute to the restriction of carrier transport. Because the SG and ET models were optimized separately, the present calculations describe their intrinsic electronic characteristics rather than the detailed charge transfer or interaction geometry at an explicit SG-ET interface. Under an applied electric field, these low-energy electron-trapping states may preferentially capture free electrons injected from the electrodes or generated within the material, thereby reducing the mean free path and mobility of charge carriers and restricting further charge injection and long-range carrier transport. Therefore, the calculations provide electronic-structure support for the proposed carrier-trapping mechanism underlying the enhanced insulating properties of the composites.

Figure 2h,i shows the surface electrostatic potential (ESP) distributions of the separately optimized ET molecule and representative silicone gel chain segment. Compared with the SG chain-segment model, ET exhibited a more heterogeneous ESP distribution, with distinct distribution peaks in the ranges of approximately −10 and 15–20 kcal/mol. This result indicates stronger localized polarity and a more pronounced dipole distribution within the isolated ET molecule. In addition, the larger positive ESP area suggests a stronger electrophilic character of ET [39]. These molecular characteristics indicate that ET may participate in electrostatic interactions with surrounding siloxane segments after dispersion in the silicone gel matrix and may contribute to its dielectric response and electron-accepting behavior. Because no explicit SG-ET complex was included in the present calculations, the detailed interaction geometry, interfacial charge redistribution, and local electric-field modification cannot be directly determined from the current molecular models.

The representative SG chain segment captures the principal local chemical structure of the silicone matrix but does not reproduce the complete crosslinked and conformationally heterogeneous gel network. Therefore, the calculated orbital energies and ESP characteristics are interpreted as qualitative indicators of the intrinsic electronic properties of SG and ET rather than as quantitative descriptions of the actual silicone gel network.

### 2.4. Dielectric Property Evolution of SG-ET Composites

**Figure 3:**
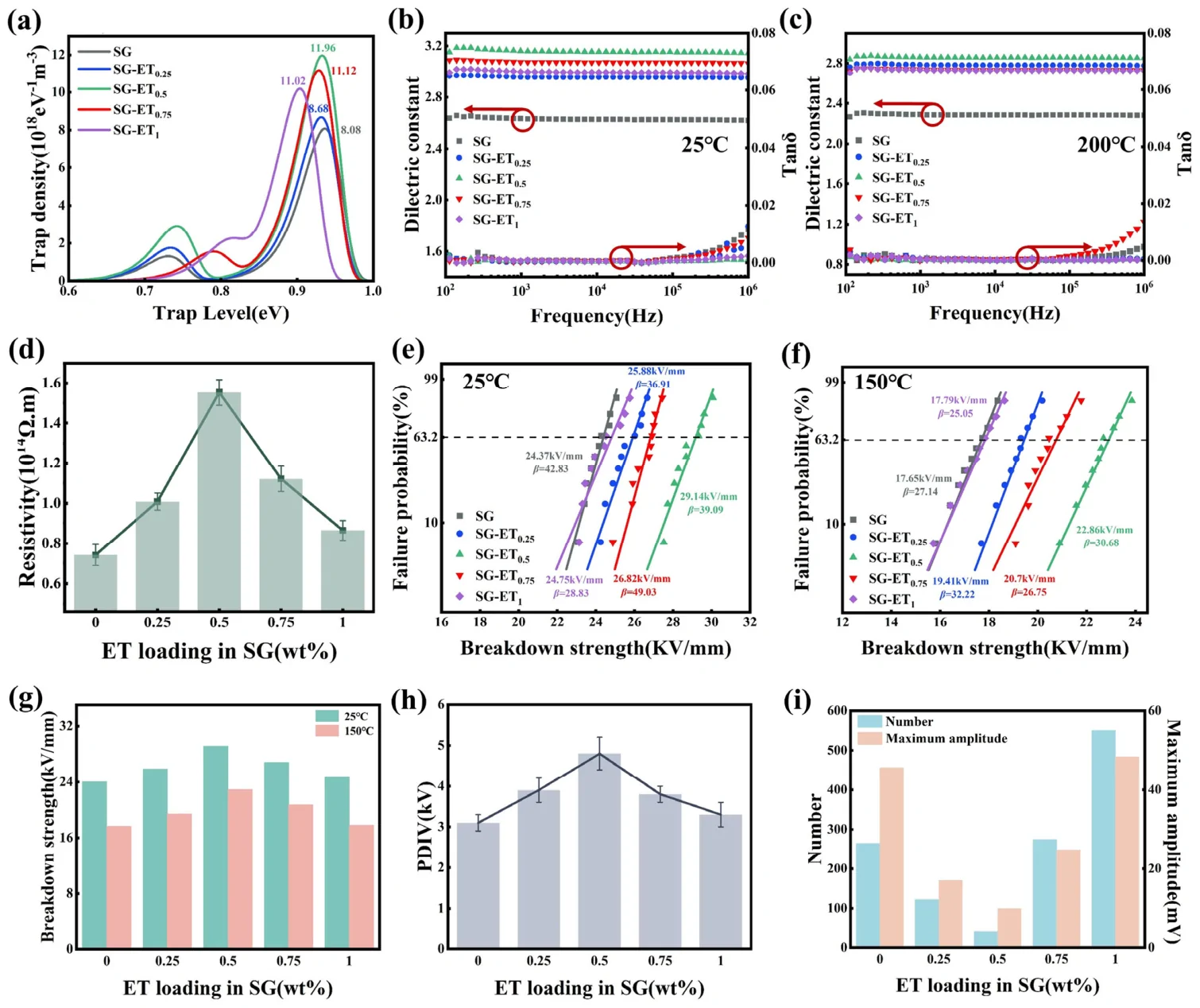
Electrical characterization of SG-ET composites. (**a**) Trap energy-level profiles obtained from SPD analysis. Frequency-dependent relative permittivity and dielectric loss measured at (**b**) 25 °C and (**c**) 200 °C. (**d**) Variation in volume resistivity with ET content. Weibull plots of AC breakdown strength at (**e**) 25 °C and (**f**) 150 °C. (**g**) Comparison of breakdown strength under room-temperature and elevated-temperature conditions. (**h**) Partial discharge inception voltage of different composites. (**i**) Statistical analysis of partial discharge events and maximum discharge amplitude after applying 7 kV AC voltage for 600 s.

To clarify how ET regulates charge transport, the trap energy-level distributions and corresponding trap densities of SG-ET composites were analyzed, as presented in Figure 3a. All samples exhibited obvious bimodal distributions, corresponding to shallow traps at 0.70–0.80 eV and deep traps at 0.85–0.95 eV. In general, shallow traps mainly influence charge migration and release processes, whereas deep traps provide more stable capture sites for injected charges, thereby restricting subsequent carrier transport and charge redistribution. Therefore, both shallow and deep traps jointly affect the insulating properties of the material [40].

The trap density exhibited a nonmonotonic dependence on ET content, with an initial increase followed by a decline at higher loadings. For SG-ET_0.5_, both the shallow-trap and deep-trap peak densities reached their maximum values, and the deep-trap density increased from 8.08 × 10^18^ eV^−1^m^−3^ for pristine SG to 1.196 × 10^19^ eV^−1^m^−3^. The increased density of both shallow and deep traps suggests that moderate ET incorporation provided additional effective charge-trapping centers, thereby improving the carrier-capture capability of the composite system. This phenomenon may be related to the localized interfacial regions formed after ET incorporation and the induced electronic-structure inhomogeneity, which is consistent with the molecular simulation results.

Notably, when the ET content was further increased to 1 wt%, the deep-trap peak position decreased from 0.95 to 0.89 eV, showing a shift toward the lower-energy region. One possible explanation is that localized ET aggregation or microcrystalline ordering at the higher loading may alter the local structural environment and trap distribution, which could weaken the carrier-binding capability of the associated trapping sites and limit further improvement in insulation performance.

The SPD analysis derives the trap energy and trap density distributions from surface-potential relaxation and therefore provides experimental evidence of changes in the trap characteristics after ET incorporation. Nevertheless, this method does not directly resolve the spatial distribution of charges within the material. TSDC and space-charge measurements would provide complementary information on trap-release dynamics and internal charge accumulation and will be considered in future investigations.

Relative permittivity (*ε_r_*) and dielectric loss (*tanδ*) are important parameters for characterizing the insulating properties of materials [41,42]. The relative permittivity and dielectric loss curves of pristine SG and SG-ET composites with different ET contents over the frequency range of 10^2^–10^6^ Hz at 25 °C and 200 °C are shown in Figure 3b,c. All samples exhibited stable polarization responses throughout the tested frequency range. With increasing ET content, the relative permittivity of the composites showed a nonlinear trend of first increasing and then decreasing. When the ET content reached 0.5 wt%, SG-ET_0.5_ exhibited the highest permittivity at both 25 °C and 200 °C. Specifically, at 1 kHz and 25 °C, the relative permittivity increased from 2.63 for pristine SG to 3.16 for SG-ET_0.5_, while a value of 2.85 was maintained at 200 °C.

This enhancement may be associated with the combined contributions of ET-related dipolar polarization and SG-ET interfacial polarization. Although the C=O absorption band of SG-ET_0.5_ is not independently resolved in the FTIR spectrum because of its ultralow ET content, the incorporated ET molecules intrinsically contain polar carbonyl groups that can respond to the applied electric field. At the moderate loading of 0.5 wt%, ET is expected to be relatively well dispersed within the silicone gel network, allowing its molecular dipoles and the associated SG-ET interfacial regions to effectively contribute to the polarization response. Consequently, SG-ET_0.5_ exhibits the highest relative permittivity among the compositions investigated. According to Equation (1), the electric field intensity *E* at the triple-junction region can be simply expressed as follows [43]:

(1) $$E=\frac{U}{d_{1}+d_{2}\frac{\varepsilon_{1}}{\varepsilon_{2}}}$$

where *U* is the applied voltage; *d*_1_ and *d*_2_ are the thicknesses of the silicone gel and ceramic substrate, respectively. *ε*_1_ and *ε*_2_ are the relative permittivities of the silicone gel and ceramic substrate, respectively. These results suggest that increasing the relative permittivity of silicone gel is beneficial for alleviating localized electric-field concentration at the triple-junction region in high-voltage devices.

However, the relative permittivity did not further increase when the ET content was raised to 0.75 and 1 wt%, but instead decreased slightly. This indicated that the regulation of the SG relative permittivity by ET was not simply content-dependent. Although the ET-related FTIR bands became more discernible at 0.75 and 1 wt% because of the increased ET concentration, this does not necessarily correspond to a stronger effective dielectric polarization. Excess ET may induce local aggregation or microcrystallization within the matrix, which reduces the number of effectively responding interfaces and restricts the orientational response of the polar units under an applied electric field. Consequently, the relative permittivity decreases at higher ET contents.

The dielectric loss results further reflected the effect of ET incorporation on the stability of the AC response. At 25 °C, all samples maintained a low loss level over a wide frequency range, indicating that ET incorporation did not significantly increase conductive loss or trigger strong dipole relaxation. In contrast, the dielectric loss of the samples at 200 °C increased to some extent in the high-frequency region, which was mainly related to enhanced silicone gel chain-segment motion, intensified thermal excitation of charge carriers, and strengthened interfacial polarization relaxation at elevated temperature. However, SG-ET_0.5_ still exhibited relatively low and stable dielectric loss, indicating that the deep traps formed by an appropriate amount of ET could effectively capture thermally excited carriers and injected charges and suppress their long-range migration, thereby avoiding a significant increase in dielectric loss. In comparison, samples with higher ET content exhibited increased dielectric loss at high temperature and high frequency. This behavior may be associated with increased local dielectric heterogeneity arising from possible ET enrichment or aggregation, which could enhance the nonuniformity of the local polarization response and the associated dielectric dissipation. Overall, these results demonstrated that an appropriate ET content improved the relative permittivity without sacrificing the low-loss characteristics, which is important for enhancing the dielectric stability of encapsulation insulating materials under high-field conditions.

Volume resistivity reflects the resistance of dielectric materials to leakage-current formation and charge-carrier transport. Figure 3d shows the volume resistivity of pristine SG and SG-ET composites with different ET contents. The volume resistivity of the composites showed a trend of first increasing and then decreasing with increasing ET content. The resistivity of pristine SG was 7.5 × 10^13^ Ω·m. When the ET content increased to 0.5 wt%, the resistivity of SG-ET_0.5_ reached a maximum value of 1.55 × 10^14^ Ω·m, nearly twice that of pristine SG. As the ET content was further increased to 0.75 and 1 wt%, the resistivity began to decrease, although that of SG-ET_1_ remained slightly higher than that of pristine SG. This variation trend was highly consistent with the trap distribution results. The improved volume resistivity at moderate ET loading indicates more effective suppression of charge transport, which is beneficial for enhancing the breakdown performance of the composites [44].

To evaluate the macroscopic effect of ET incorporation on the insulating properties of silicone gel, the AC breakdown strengths of different samples were tested at 25 and 150 °C. The Weibull distribution results are shown in Figure 3e,f. Among all samples, SG-ET_0.5_ exhibited the highest breakdown strength of 29.14 kV/mm at room temperature, which was 19.57% higher than that of pristine SG, 24.37 kV/mm, demonstrating the best insulation-enhancement effect.

Figure 3g compares the breakdown strengths of different samples at 25 and 150 °C. Under the elevated temperature condition of 150 °C, the breakdown strength of all samples decreased compared with that at 25 °C. Nevertheless, SG-ET_0.5_ still maintained the optimal performance. The breakdown strength of pristine SG decreased to 17.52 kV/mm at 150 °C, corresponding to a reduction of 28.1%, whereas that of SG-ET_0.5_ decreased to 22.86 kV/mm, corresponding to a smaller reduction of 21.56%.

Combined with the DFT calculations and SPD results, it can be inferred that the lower LUMO energy level and stronger electron affinity of ET molecules introduced effective electron-capturing sites into the silicone gel matrix. These deep traps can preferentially capture high-energy electrons generated by electrode injection or thermal excitation, reduce carrier mobility and mean free path, and thus delay the development of impact ionization and breakdown channels. At 150 °C, enhanced polymer chain-segment motion, increased free volume, and a larger number of thermally excited carriers usually accelerate charge transport and reduce breakdown strength. However, SG-ET_0.5_ still exhibited significantly enhanced breakdown performance, indicating that ET-induced deep-trap capture and localized polar interactions can partly counteract the adverse effects of high temperature on insulation stability. Therefore, the introduction of an appropriate amount of ET provides an effective approach for improving the high-temperature breakdown performance and insulation stability of silicone gel under the investigated conditions.

Partial discharge tests were performed to comparatively evaluate the effect of ET on the partial-discharge inception and discharge behavior of silicone gel under a strongly nonuniform electric field. The partial discharge inception voltage (PDIV) of samples with different ET contents was first measured. Subsequently, an overvoltage level of 7 kV, approximately corresponding to 1.5 times the maximum PDIV, was applied for 600 s, during which the number of partial discharge events and the maximum discharge amplitude were recorded [45,46]. The PDIV results are shown in Figure 3h. Pristine SG exhibited the lowest PDIV of approximately 3.1 kV, indicating that it was more susceptible to partial discharge under strongly nonuniform electric fields. After ET incorporation, the partial-discharge resistance of the composites was significantly improved. SG-ET_0.5_ exhibited the best performance, with the PDIV increasing to 4.8 kV, corresponding to a substantial improvement of approximately 54.8% compared with pristine SG. When the ET content was further increased to 0.75 and 1 wt%, the PDIV decreased to approximately 3.8 and 3.3 kV, respectively, and the enhancement effect was significantly weakened. These results indicated that the regulation of the partial discharge inception process in silicone gel by ET exhibited significant concentration dependence, and excessive ET was unfavorable for maintaining this advantage.

The partial-discharge time-domain profiles of each composite under an overvoltage condition of 7 kV for 600 s are shown in Figure S1a–e. Pristine SG exhibited intense discharge activity under the sustained high electric field, and the discharge pulse amplitude fluctuated markedly in the time-domain profiles. This indicated that charge acceleration and impact ionization were more likely to occur in pristine SG within regions of localized electric-field concentration, further developing into stable discharge channels. In contrast, the discharge behavior of the composites was significantly suppressed after ET incorporation, as evidenced by sparser time-domain distributions and markedly reduced discharge amplitudes.

As shown in Figure 3i, compared with the maximum discharge amplitude of 46 mV for pristine SG, SG-ET_0.5_ exhibited the best performance, with the discharge amplitude mostly controlled below 10 mV. Moreover, the total number of discharge events decreased from 270 for pristine SG to 45 for SG-ET_0.5_. These results indicate that the introduction of an appropriate amount of ET not only increases the partial discharge inception voltage but also suppresses discharge activity under the investigated 7 kV overvoltage condition, as evidenced by the reduced number of discharge events and maximum discharge amplitude. However, when the ET content was further increased to 1 wt%, the partial-discharge performance deteriorated significantly, and the total number of discharge events increased to approximately 550 within 600 s, even higher than that of pristine SG.

Based on the preceding results, increasing the ET content above 0.5 wt% may promote localized ET enrichment, aggregation, or microcrystalline ordering within the silicone gel matrix. These possible structural changes could increase local dielectric and microstructural heterogeneity and may consequently weaken the insulation-enhancement effect achieved at the moderate ET loading.

**Figure 4:**
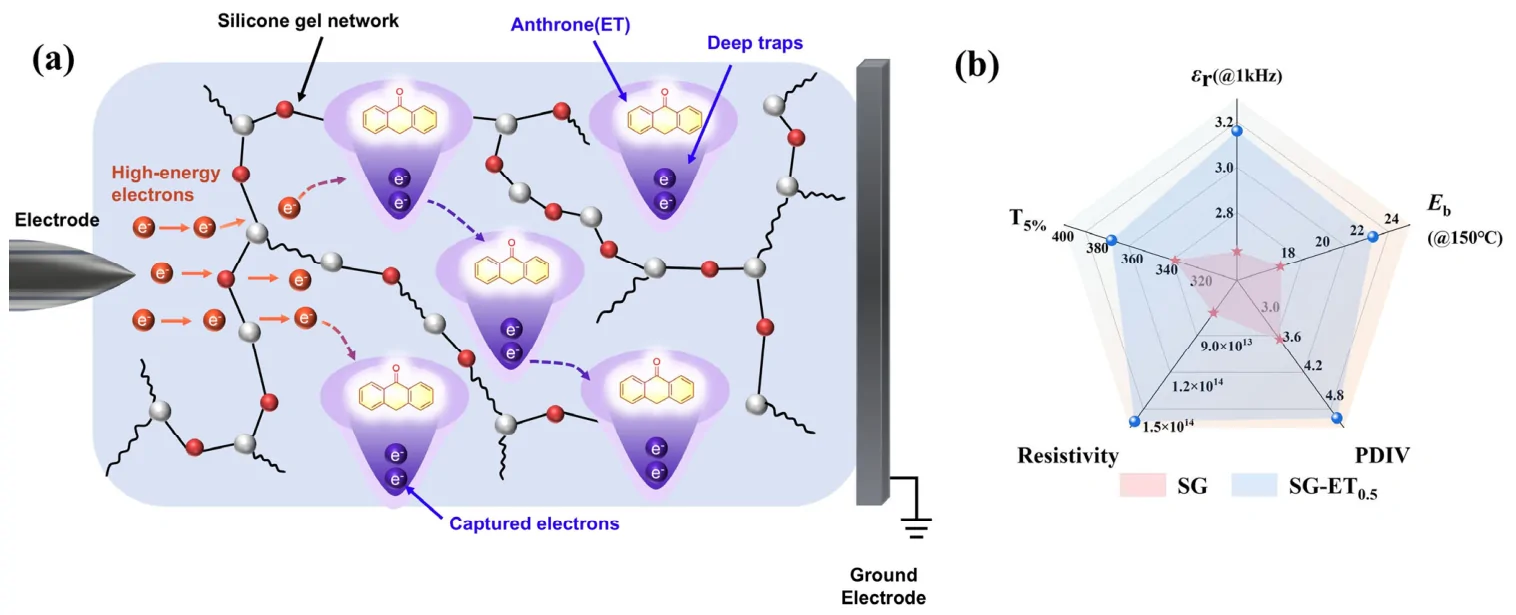
(**a**) Schematic illustration of the dielectric performance enhancement mechanism of the composites. (**b**) Radar chart comparing the key performance parameters of SG and SG-ET_0.5_.

The significant enhancement in partial-discharge performance may be associated with the combined effects of the experimentally observed trap distribution and the molecular characteristics of ET. Under low-loading conditions, the increased deep-trap density may favor the capture of high-energy electrons injected from the electrodes, potentially reducing their mean free path and inhibiting impact ionization. This process may hinder the formation of electron avalanches and slow the development of streamer-like discharge channels. Meanwhile, the aromatic conjugated structure of ET molecules may further contribute to discharge suppression by delocalizing the energy of high-energy electrons and weakening collision-induced excitation. These combined effects may account for the optimal PDIV, discharge amplitude, and number of discharge events observed for SG-ET_0.5_, as illustrated in Figure 4a. The present 600 s overvoltage test provides a comparative evaluation of partial-discharge behavior among different SG-ET formulations under identical accelerated laboratory conditions. A systematic assessment of long-term service durability requires extended partial-discharge exposure and electro-thermal aging studies.

Finally, the performance parameters of SG and SG-ET_0.5_ were compared, as shown in Figure 4b. Compared with pristine SG, SG-ET_0.5_ exhibited higher breakdown strength at elevated temperature, improved thermal stability, and enhanced key electrical properties, including volume resistivity, relative permittivity, and PDIV. Therefore, SG-ET_0.5_ demonstrated superior overall performance and shows promising potential as a soft encapsulation dielectric for high-voltage power modules.

All samples were vacuum-dried before characterization, and the present results therefore represent the relative thermal and electrical properties of the SG-ET composites under controlled dry conditions. The effects of moisture absorption and controlled humidity exposure on dielectric response, charge transport, breakdown strength, and partial-discharge behavior require further systematic investigation.

Ceramic fillers such as h-BN are generally effective in improving the thermal conductivity of silicone materials, but relatively high filler loadings are often required, which may increase viscosity and introduce additional filler–matrix interfaces [24]. SiC and BaTiO3 can regulate dielectric response and charge transport, whereas their modification effects strongly depend on filler content, dispersion uniformity, and interfacial compatibility [21]. Molecular-level modification using glycerol or phenyl groups can avoid the introduction of large amounts of inorganic interfaces, but may influence the crosslinked network, curing behavior, or preparation complexity [30,31]. Owing to differences in material formulations, sample dimensions, electrode configurations, and testing conditions, the absolute property values reported in these studies are not directly comparable. In the present study, an ultralow ET loading of 0.5 wt% simultaneously improved the breakdown strength, volume resistivity, PDIV, and thermal stability of silicone gel, while maintaining a viscosity nearly identical to that of pristine SG. Therefore, the principal advantage of the ET-based strategy lies in achieving a favorable balance among electrical insulation, thermal stability, ultralow additive loading, and processing adaptability.

## 3. Conclusions

In this study, an ultralow-loading organic molecular semiconductor strategy was proposed to improve the insulating properties and thermal stability of silicone gel. By introducing ET into the silicone gel matrix, the dielectric performance, breakdown strength, partial-discharge resistance, and thermal stability of silicone gel were simultaneously enhanced while maintaining favorable flowability and processing compatibility. At the optimal ET loading of 0.5 wt%, SG-ET_0.5_ exhibited a breakdown strength of 29.14 kV/mm at 25 °C, representing a 19.57% improvement compared with pristine SG. Even at 150 °C, the breakdown strength decreased by only 21.56%, indicating superior high-temperature insulation stability. Meanwhile, SG-ET_0.5_ showed an increased relative permittivity of 3.16 at 1 kHz while maintaining low dielectric loss. Its PDIV increased to 4.8 kV, accompanied by a reduced discharge amplitude of less than 10 mV under overvoltage conditions.

The enhanced electrical insulation performance may be associated with the combined effects of deep-trap regulation and the molecular structural characteristics of ET. The low-lying LUMO level, polar carbonyl group, and aromatic conjugated structure of ET may facilitate the capture of high-energy electrons and thereby contribute to suppressed electron avalanche development, restricted charge transport, and reduced leakage current. In addition, SG-ET_0.5_ maintained excellent thermal stability, while preserving good flowability. These results demonstrate that organic molecular semiconductor-enabled deep-trap engineering is an effective low-loading strategy for improving the electrical insulation performance of soft encapsulation dielectrics without sacrificing processing compatibility, providing molecular-level guidance for next-generation power electronic packaging insulation materials. Future work will focus on extended partial-discharge exposure, electro-thermal aging, moisture-absorption measurements, and controlled humidity testing to clarify the evolution of insulation performance and provide an experimental basis for evaluating the long-term durability, environmental stability, and service life of the composites.

## 4. Materials and Methods

### 4.1. Reagents

The 195T A/B silicone gel used in this work was supplied by Shenzhen Ausbond Co., Ltd. China, with an A/B mass ratio of 10:1. Component A was vinyl-terminated methyl silicone oil, and component B was side-chain hydrogen-containing methyl silicone oil. The molecular crosslinking reaction of silicone gel is shown in Figure S2. Anthrone, a light-yellow crystalline organic molecular semiconductor, was purchased from Macklin Co., Ltd. China, and its molecular structure is shown in Figure S3.

### 4.2. Preparation of SG-ET Composites

**Figure 5:**
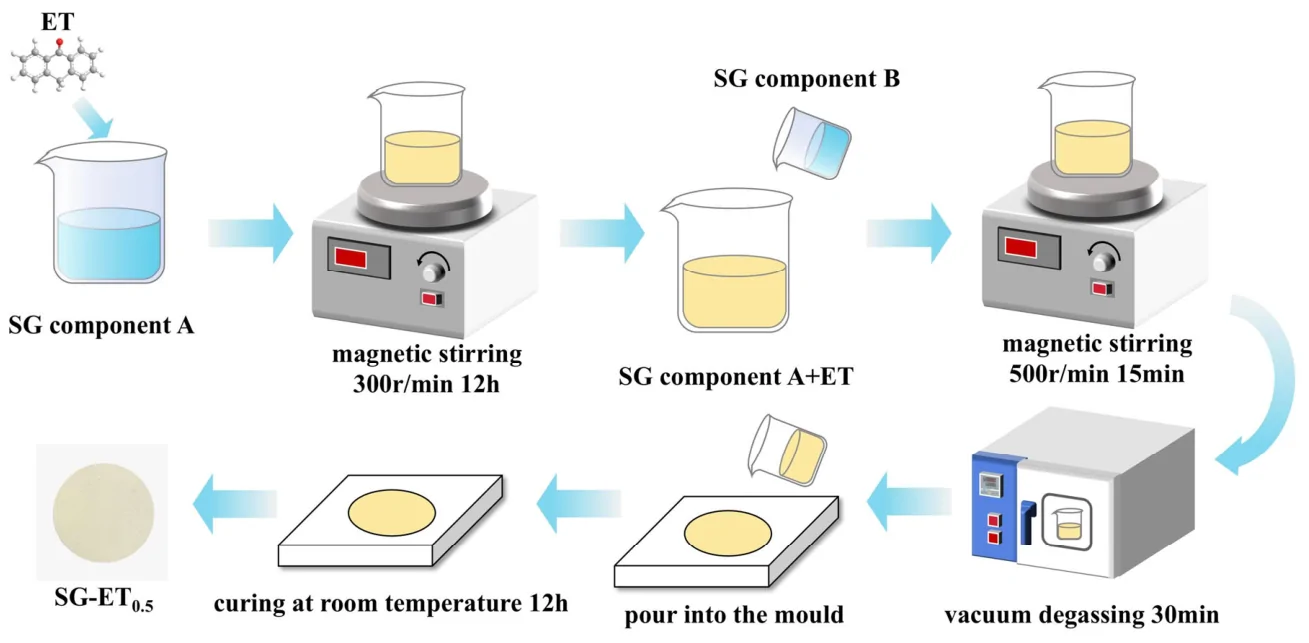
The preparation process for composites with varying ET contents.

The fabrication route for the SG-ET composites is schematically shown in Figure 5. First, ET was weighed according to the designed mass fraction. A certain amount of silicone gel component A and ET was added into a beaker and stirred at 300 rpm for 12 h to ensure uniform dispersion. Subsequently, silicone gel component B was added to the mixture, followed by vigorous stirring for 15 min. After vacuum degassing for 30 min, the mixture was poured into a PTFE mold and cured at room temperature for 12 h. The composites with ET mass fractions of 0, 0.25, 0.5, 0.75, and 1 wt% were prepared and denoted as SG, SG-ET_0.25_, SG-ET_0.5_, SG-ET_0.75_, and SG-ET_1_, respectively. The as-prepared samples are shown in Figure 1c.

### 4.3. Experimental Method

#### 4.3.1. Microstructural Characterization

The chemical structures of SG and SG-ET composites were characterized by Fourier transform infrared spectroscopy (FTIR, VERTEX 80V, Bruker, Ettlingen, Germany) over the wavenumber range of 4000–400 cm^−1^. X-ray diffraction (XRD) patterns of the samples and ET crystals were recorded using a SmartLab diffractometer (9kW, Rigaku Co., Ltd., Tokyo, Japan) in the 2θ range of 5–60°. The cross-sectional and surface morphologies of the composites were observed using scanning electron microscopy (SEM, GAIA3Ga, TESCAN, Brno, Czech Republic).

#### 4.3.2. Mechanical and Thermal Performance Testing

The thermal stability of the samples was evaluated by thermogravimetric analysis (TGA, STA200, HITACHI, Tokyo, Japan) under nitrogen from room temperature to 800 °C at a heating rate of 10 °C/min. The viscosity of the uncured composite solutions was measured using a DV3T viscometer (Brookfield, MA, USA) to assess their flowability and applicability for power electronic encapsulation. Thermal conductivity and thermal diffusivity were tested at room temperature using an HFM436 instrument (NETZSCH-Gerätebau GmbH, Selb, Germany). The coefficient of thermal expansion (CTE) was measured using a TMA Q400EM analyzer (TA Instruments, New Castle, DE, USA) over the temperature range of 25–250 °C at 10 °C/min, and the CTE values were determined by linear fitting of the dimensional change-temperature curves.

#### 4.3.3. Molecular Simulation

In this study, an isolated ET molecule and a representative SG chain segment were constructed as separate molecular models. The SG chain segment retains the dominant Si-O-Si backbone and methyl-substituted local chemical environment of silicone gel and was used to qualitatively represent its intrinsic electronic characteristics. Their geometries were independently optimized using Gaussian 16W A.03 based on density functional theory with the B3LYP functional. No explicit SG-ET complex or interfacial configuration was constructed. Because the actual silicone gel is a three-dimensional crosslinked and conformationally disordered network, the simplified SG model does not reproduce the complete crosslinking topology, chain-length distribution, intermolecular packing, or dynamic chain-segment motion of the real material. The independently optimized structures were therefore used for qualitative comparison of the frontier molecular orbital energy levels and molecular surface electrostatic-potential distributions. Quantitative analysis of the molecular surface electrostatic potentials was subsequently performed using the Multiwfn 2026.3.18 program.

#### 4.3.4. Electrical Performance Testing

Before thermal and electrical characterization, all samples were vacuum-dried to minimize the influence of residual and environmentally absorbed moisture. The dried samples were subsequently stored under sealed conditions before testing. The microscopic charge transport behavior of the samples was characterized by surface potential decay (SPD) measurements. The experimental setup is illustrated in Figure S4. During the measurement, each sample was first placed 5 mm below the corona needle electrode and charged at 10 kV for 15 min. Subsequently, the charged sample was moved to a position 3 mm below the electrostatic probe, and the surface potential decay was recorded for 40 min after removing the applied voltage. The obtained SPD curves were further used to calculate the trap energy level and trap density [47].

A Novocontrol Concept 80 (Montabaur, Germany) dielectric spectrometer was employed to measure the dielectric response of SG and SG-ET composites at 25 °C and 200 °C. As shown in Figure S5, the test platform consisted of an Alpha-A impedance analyzer, a temperature-controlled chamber, and a computer control system. The relative permittivity and dielectric loss were recorded over the frequency range of 10^2^–10^6^ Hz.

The volume resistivity of the samples was measured using a three-electrode system connected to a Keithley 6514 electrometer. As shown in Figure S6, the system consisted of a positive DC high-voltage power supply, a three-electrode fixture, a protective resistor, a shielding box, and the electrometer. A DC voltage of 10 kV was applied for 40 min, and the corresponding leakage current was recorded. Each sample was tested five times, and the average value was used. The volume resistivity was calculated using Equation (2):

(2) $$\rho =\frac{US}{IL}$$

where *U* is the applied voltage, *S* is the sample area, *I* is the leakage current, and *L* is the sample thickness.

The breakdown strength of the samples was tested at 25 °C and 150 °C using a parallel-plate electrode configuration. The PTFE fixture contained plate electrodes with a diameter of 25 mm and a chamfer radius of 2.5 mm. An AC voltage was applied at a ramp rate of 1 kV/s until electrical breakdown occurred. For each sample, ten valid breakdown data points were collected and analyzed using the Weibull distribution model according to Equation (3) [48]:

(3) $$P\left(E\right)=1-exp\left[-{\left(\frac{E}{\alpha }\right)}^{\beta }\right]$$

(4) $$E=\frac{U}{d}$$

where *E* is the breakdown strength and *P(E)* denotes the cumulative probability of the corresponding parameter *E*. *α* is the scale parameter and *β* is the shape parameter. *U* in Equation (4) is the breakdown voltage of the composite, and *d* is the sample thickness.

A needle-plate electrode partial discharge test platform was established, as illustrated in Figure S7. The SG sample was encapsulated in a mold with a pre-embedded needle electrode to simulate local electric-field distortion at the needle tip. During testing, AC voltage was applied to the needle electrode, and the partial discharge signals were collected using a high-frequency current transformer (HFCT) sensor. The partial discharge inception voltage (PDIV) of each composite was determined. In addition, the number of partial discharge events and the corresponding discharge amplitudes were recorded under an overvoltage condition of 7 kV for 600 s. The overvoltage test was used for the comparative evaluation of partial-discharge behavior among the different compositions under identical laboratory conditions.

## Data Availability

Data is contained within the article. Further inquiries can be directed to the corresponding author.

## Supplementary Materials

The following supporting information can be downloaded at: https://www.mdpi.com/article/10.3390/gels12080668/s1, Figure S1: (a–e) Time-domain partial discharge signals of the composite material over 600 s; Figure S2: Crosslinking reaction between components A and B of silicone gel; Figure S3: Molecular structure of Anthrone; Figure S4: Surface potential decay (SPD) testing platform; Figure S5: Dielectric property testing platform; Figure S6: Three-electrode resistivity testing platform; Figure S7: Partial discharge testing platform.
