# Supplementary material for: Ultralow-Loading Anthrone Molecular Semiconductor for Enhancing the Insulation Reliability of Silicone Gel Dielectrics
Source: Gels. 2026 Jul 25;12(8):668. doi: 10.3390/gels12080668 (PMC13512122; doi:10.3390/gels12080668)
Supplement: Supplementary file 1 [file gels-12-00668-s001.zip › gels-4455114-supplementary.pdf]

Ultralow-Loading Anthrone Molecular Semiconductor for Enhancing the Insulation Reliability of Silicone Gel Dielectrics

Mengjia Feng, Chaoyue Zhao, Wenbo Li, Zichen Cui, and Jianzeng Guo.

Supplementary Information

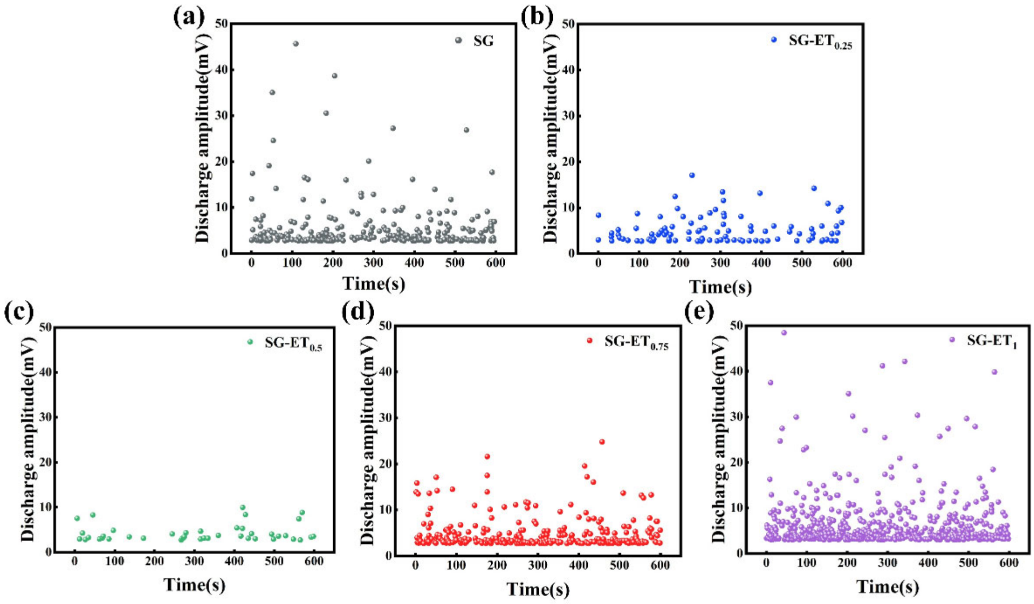

Figure S1. (a)-(e) Time-domain partial discharge signals of the composite material over 600 s

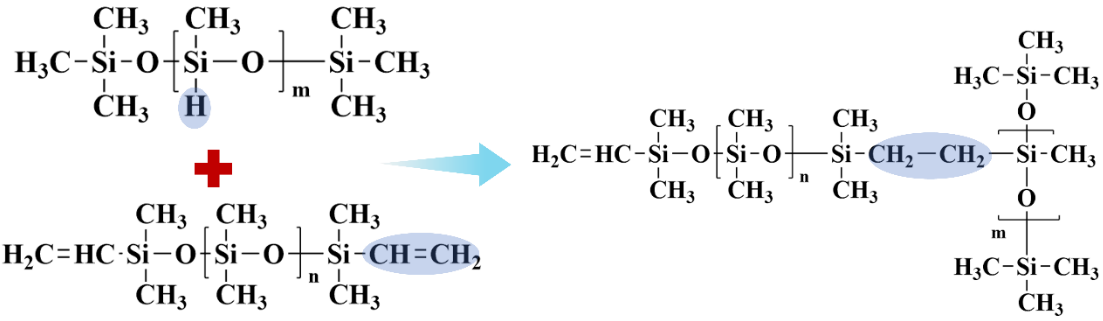

Figure S2. Crosslinking reaction between components A and B of silicone gel

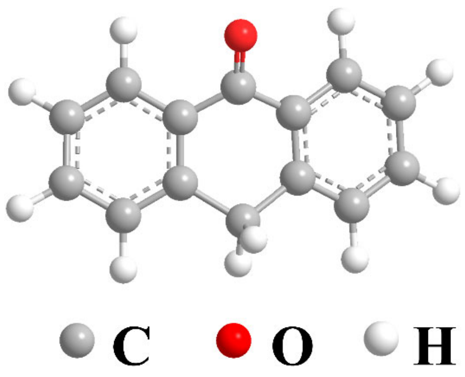

Figure S3. Molecular structure of Anthrone

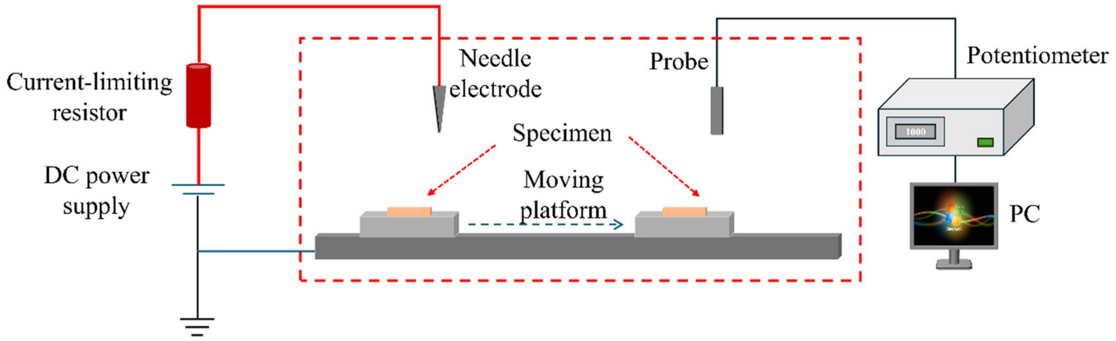

Figure S4. Surface potential decay (SPD) testing platform

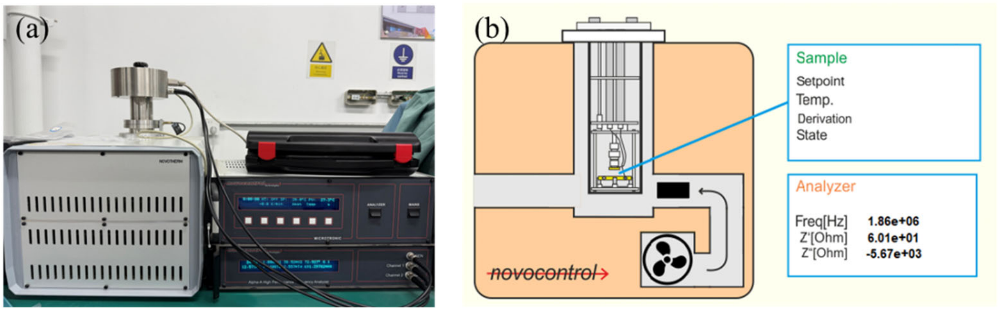

Figure S5. Dielectric property testing platform

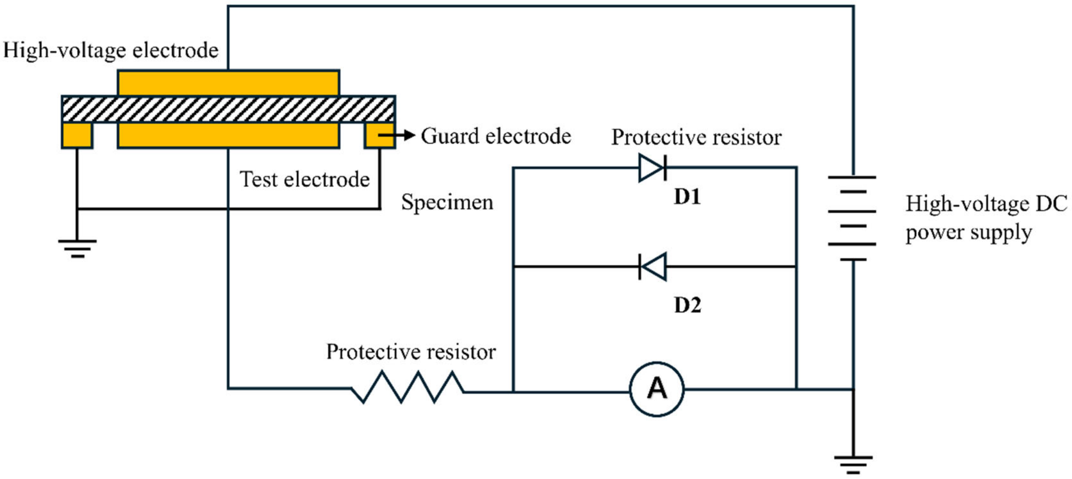

Figure S6. Three-electrode resistivity testing platform

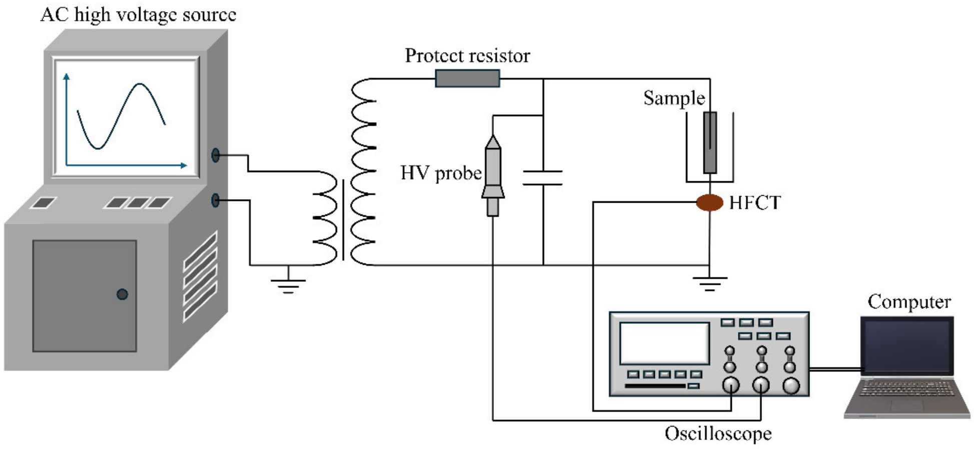

Figure S7. Partial discharge testing platform
